# Marine Meroterpenoids Isolated from *Gongolaria abies-marina* Induce Programmed Cell Death in *Naegleria fowleri*

**DOI:** 10.3390/ph16071010

**Published:** 2023-07-17

**Authors:** Iñigo Arberas-Jiménez, Rubén L. Rodríguez-Expósito, Desirée San Nicolás-Hernández, Javier Chao-Pellicer, Ines Sifaoui, Ana R. Díaz-Marrero, José J. Fernández, José E. Piñero, Jacob Lorenzo-Morales

**Affiliations:** 1 Instituto Universitario de Enfermedades Tropicales y Salud Pública de Canarias, Universidad de La Laguna, Avda. Astrofísico Fco. Sánchez, S/N, 38206 La Laguna, Tenerife, Spain; iarberas@ull.edu.es (I.A.-J.); rrodrige@ull.edu.es (R.L.R.-E.); dsannico@ull.edu.es (D.S.N.-H.); alu0101016429@ull.edu.es (J.C.-P.); isifaoui@ull.edu.es (I.S.); 2 Departamento de Obstetricia y Ginecología, Pediatría, Medicina Preventiva y Salud Pública, Toxicología, Medicina Legal y Forense y Parasitología, Universidad de La Laguna, 38206 La Laguna, Tenerife, Spain; 3 Centro de Investigación Biomédica en Red de Enfermedades Infecciosas (CIBERINFEC), Instituto de Salud Carlos III, 28220 Madrid, Spain; 4 Instituto Universitario de Bio-Orgánica Antonio González, Universidad de La Laguna, Avda. Astrofísico Fco. Sánchez 3, 38206 La Laguna, Tenerife, Spain; 5 Instituto de Productos Naturales y Agrobiología (IPNA), Consejo Superior de Investigaciones Científicas (CSIC), Avda. Astrofísico Fco. Sánchez 3, 38206 La Laguna, Tenerife, Spain; 6 Departamento de Química Orgánica, Universidad de La Laguna (ULL), Avda. Astrofísico Fco. Sánchez 3, 38206 La Laguna, Tenerife, Spain

**Keywords:** meroterpenoids, marine natural products, *Gongolaria abies-marina*, *Naegleria fowleri*, meningoencephalitis

## Abstract

*Naegleria fowleri* is the causative agent of a central nervous system affecting disease called primary amoebic meningoencephalitis. It is a fulminant disease with a rapid progression that affects mainly children and young adults who report previous water exposure. Current treatment options are not totally effective and involve several side effects. In this work, six meroterpenoids isolated from the brown algae *Gongolaria abies-marina* were evaluated against *N. fowleri*. Gongolarone B (**1**), 6*Z*-1′-methoxyamentadione (**2**), and 1′-methoxyamentadione (**3**) were the most active molecules against *N. fowleri* with IC_50_ values between 13.27 ± 0.96 µM and 21.92 ± 1.60 µM. However, cystomexicone B (**6**) was the molecule with the highest selectivity index (>8.5). Moreover, all these compounds induced different cellular events compatible with the apoptosis-like PCD process, such as chromatin condensation, damages at the mitochondrial level, cell membrane disruption, and production of reactive oxygen species (ROS). Therefore, *G. abies-marin*a could be considered as a promising source of active molecules to treat the *N. fowleri* infections.

## 1. Introduction

Primary amoebic meningoencephalitis (PAM) is an acute and fulminant disease that affects the central nervous system (CNS), and it is caused by the opportunistic parasite *Naegleria fowleri*. This free-living amoeba group belonging to protozoa is capable of living in the environment, especially in warm water bodies including spas, geothermal springs, untreated and under treated domestic water supplies, and poorly maintained or untreated swimming pools [1,2]. In fact, the incidence of PAM is higher in summer months when the infective stage, the trophozoites, grow in more favorable conditions [3]. Moreover, the increase of amoebic infections in this period of the year is not exclusive to *N. fowleri* and has also been observed in other pathogenic amoebae [4].

Fowler and Carter described the first *N. fowleri* infection in 1965 and since then more than 400 cases have been described worldwide, affecting 39 countries [5]. However, the real number of cases could be higher due to misdiagnosis or unreported cases [3,5]. Moreover, there has been an increase in the number of reported cases since the year 2000, not only because of the awareness and the improvement in the diagnosis protocols and techniques but also because of the climate change which is increasing the abundance of *N. fowleri* cells as well as its geographic range [6].

PAM patients, mostly children and young adults, report previous exposure to *N. fowleri* contaminated water where the amoebae penetrate and invade the nasal cavity. After that, the trophozoites of *N. fowleri* cross the cribriform plate and invade the brain via the olfactory bulbs and the basilar brain [7]. The first symptoms, which appear within the first nine days after the infection, include severe headache, altered sense of smell, fever, or vomiting preceding stiff neck, seizures, hallucinations, coma, and death [8,9].

The uncommonness of the PAM induces a lack of information regarding the best therapeutic combination [10]. The most-used drug to treat *N. fowleri* infections is amphotericin B, an antifungal agent that is commonly administered in combination with other compounds as fluconazole, azithromycin, or rifampicin [5,11]. Moreover, miltefosine has been recently added to the spectrum of the therapeutic options against *N. fowleri* since it was used in some of the recent surviving cases [12,13]. However, these drugs are administered intravenously or intrathecally [10], leading to a poor penetration of the drugs into the CNS due to high selectivity of the blood–brain barrier [14]. Hence, high concentrations of drugs are needed, provoking the appearance of different severe side effects such as nephrotoxicity [14,15].

**Figure 1:**
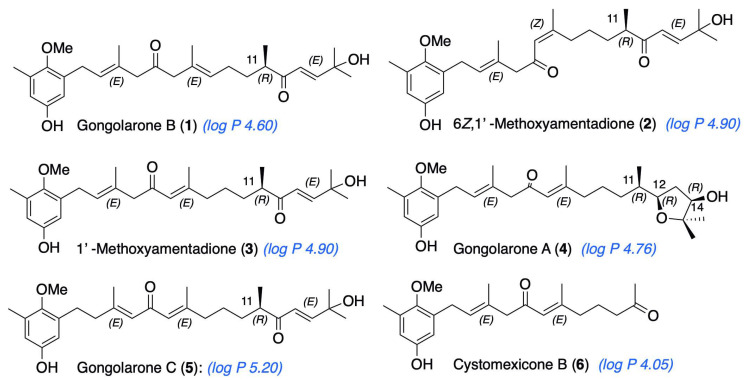
Meroterpenoids isolated from *G. abies-marina*. Calculated log *P* is indicated for each compound (ChemDraw, v. 20.1.0.112, PerkinElmer Informatics, Inc., Waltham, MA, USA.).

Within the ongoing research strategies on the study of the marine environment as a source of anti-amoebic compounds, the aim of the present work was to test in vitro the anti-*Naegleria* activity and cytotoxicity against murine macrophages of meroterpenoids 1–6 (Figure 1) isolated from the brown alga *Gongolaria abies-marina*, collected in the Canary Islands.

## 2. Results

### 2.1. Isolation and Identification of Meroterpenoids of G. abies-marina

Specimens of *G. abies-marina* were collected from the intertidal area of the northwest coast of the island of Tenerife, Canary Islands. The dried seaweed was ground and macerated with dichloromethane and ethyl acetate at room temperature to obtain a crude extract. Initially, fractionation by gel-filtration chromatography of the crude extract was carried out. Further fractionation and purification steps allowed the isolation of meroterpenoids: gongolarone B (**1**), 6*Z*-1′-methoxyamentadione (**2**), 1′-methoxyamentadione (**3**), gongolarone A (**4**), gongolarone C (**5**), and cystomexicone B (**6**). Their structures and the absolute configuration of the chiral centers were confirmed by spectroscopic analysis, biogenetic considerations, and the values of their specific rotations (Figure 1) [16].

### 2.2. In Vitro Amoebicidal Evaluation of Meroterpenoids **1**–**6** against N. fowleri

The in vitro activity evaluation of meroterpenoids **1**–**6** obtained from the *G. abies-marina* algae showed that gongolarone B (**1**), 6*Z*-1′-methoxyamentadione (**2**), and 1′-methoxyamentadione (**3**) were the most active molecules with very similar inhibitory concentration 50 (IC_50_) values against all tested strains (Table 1). On the other hand, cystomexicone B (**6**) showed also anti-*Naegleria* properties with very low cytotoxicity (cytotoxic concentration 50 (CC_50_) > 279 µM). In fact, this last compound showed the highest selectivity index value (>8.5). The in vitro activity of the compounds was also confirmed in a different *N. fowleri* strain obtaining similar IC_50_.

Due to the obtained in vitro anti-amoebic activity and the cytotoxicity values, gongolarone B (**1**), 6*Z*-1′-methoxyamentadione (**2**), 1′-methoxyamentadione (**3**), and cystomexicone B (**6**) were selected to perform the programmed cell death (PCD) induction assays.

### 2.3. Evaluation of the PCD Induction in N. fowleri

The presence of some characteristic events of the PCD, such as chromatin condensation, cell membrane disruption, increase of reactive oxygen species (ROS), and mitochondrial damages, was evaluated after the treatment of *N. fowleri* trophozoites with the inhibitory concentration 90 (IC_90_) of the selected molecules.

#### 2.3.1. Evaluation of Chromatin Condensation

**Figure 2:**
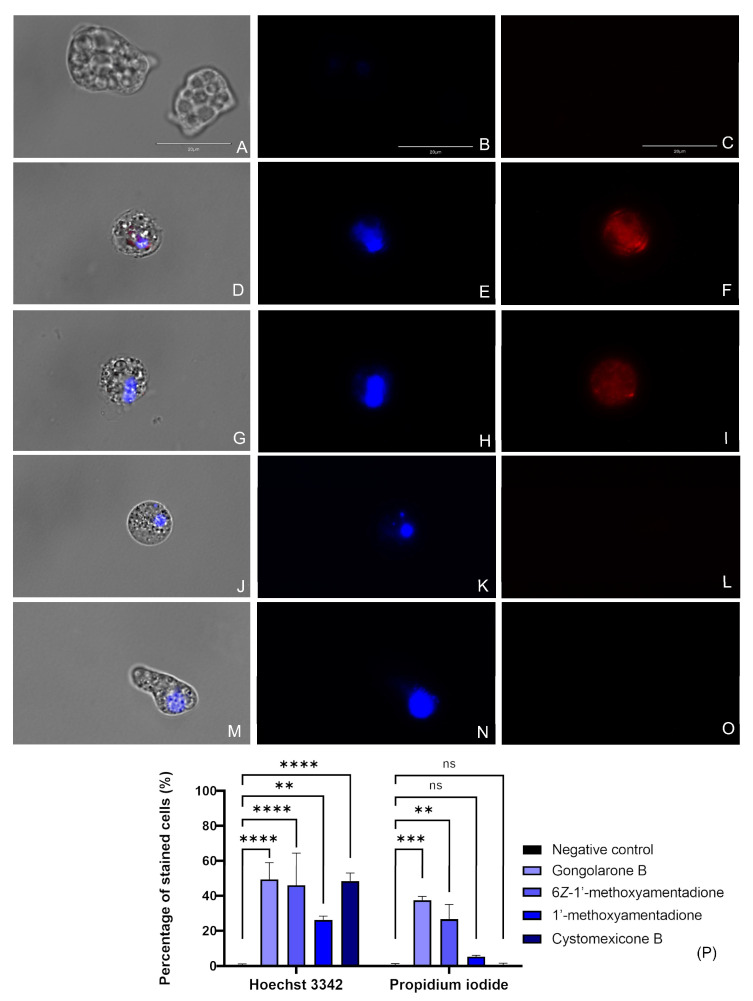
Evaluation of the presence of chromatin condensation after the incubation of the amoebae with the IC_90_ of the meroterpenoids (**D**–**O**). Negative control (**A**–**C**), gongolarone B (**1**) (**D**–**F**), 6*Z*-1′-methoxyamentadione (**2**) (**G**–**I**), 1′-methoxyamentadione (**3**) (**J**–**L**), and cystomexicone B (**6**) (**M**–**O**). Hoechst stain (**B**) and PI (**C**) show no fluorescence in non-treated cells. However, in cells treated with gongolarone B (**1**) and 6*Z*-1′-methoxyamentadione (**2**) a bright blue (Hoechst) (**E** and **H**) and red (PI) (**F** and **I**) fluorescence can be observed, suggesting that these cells are undergoing a late apoptotic phase. Moreover, cells treated with 1′-methoxyamentadione (**3**) and cystomexicone B (**6**) emitted blue fluorescence corresponding to the Hoechst stain (**K**,**N**) while no red fluorescence can be observed in the PI channel. This suggests that cells treated with 1′-methoxyamentadione (**3**) and cystomexicone B (**6**) were undergoing an early apoptotic phase. Images (×100) are representative of the cell population observed in the performed experiments. An EVOS M5000 Cell Imaging System, Life Technologies, Madrid, Spain was used to capture the images. Scale bar: 20 μm. (**P**) The bar graph shows the percentage of cells that emit fluorescence after the incubation with the Hoechst and PI dyes. Data represent the mean values of three different assays and the standard deviation (SD). A one-way analysis of variance (ANOVA) was also assessed to determine the statistical differences between the treated cells and the negative control, ** *p* < 0.01; *** *p* < 0.001; **** *p* < 0.0001; ns = not significant. For each counting, five different pictures were analyzed in the EVOS M5000 Cell Imaging System, Life Technologies.

The treatment of *N. fowleri* cells with the IC_90_ of the gongolarone B (**1**), 6*Z*-1′-methoxyamentadione (**2**), 1′-methoxyamentadione (**3**), and cystomexicone B (**6**) induced DNA condensation, as can be observed in Figure 2 and Figure S1. Treated cells show a bright blue stained nuclei corresponding to the condensed chromatin (Figure 2E,H,K,N). Furthermore, cells treated with the gongolarone B (**1**) (Figure 2F) and 6*Z*-1′-methoxyamentadione (**2**) (Figure 2I) also showed red fluorescence meaning that the propidium iodide (PI) reached the nuclei and that the cells were dead. These results suggest that cells treated with gongolarone B (**1**) and 6*Z*-1′-methoxyamentadione (**2**) were undergoing a late apoptotic phase. On the other hand, cells treated with 1′-methoxyamentadione (**3**) and cystomexicone B (**6**) showed no red fluorescence, suggesting that the amoebae were going through an early apoptotic phase.

#### 2.3.2. Plasma Membrane Permeability

**Figure 3:**
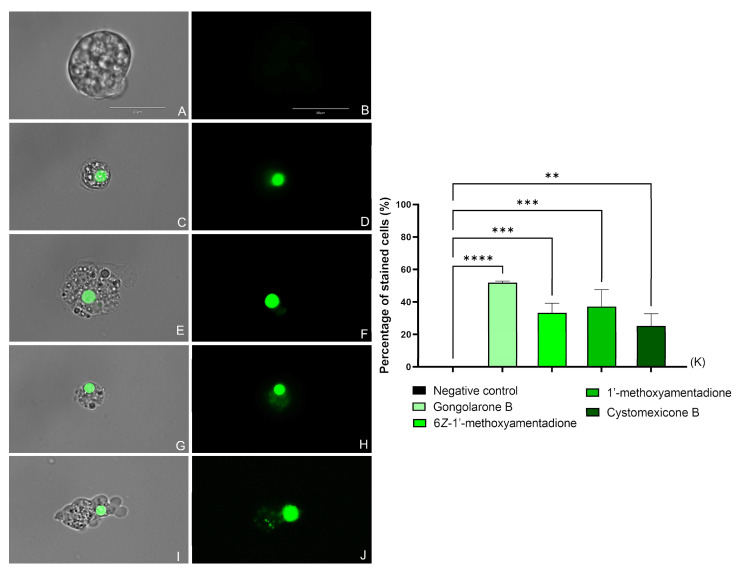
Effect of the IC_90_ of the evaluated compounds on the plasma membrane permeability of *N. fowleri* after 24 h. Negative Control (**A**,**B**), gongolarone B (**1**) (**C**,**D**), 6*Z-*1′-methoxyamentadione (**2**) (**E**,**F**), 1′-methoxyamentadione (**3**) (**G**,**H**), and cystomexicone B (**6**) (**I**,**J**). Images (×100) are representative of the cell population observed in the performed experiments. An EVOS M5000 Cell Imaging System, Life Technologies, Madrid, Spain was used to capture the images. Scale bar: 20 µm. (**K**) The graph includes the mean percentage ± SD of the stained cells. Each experiment was conducted on three different days. For each counting, five different pictures were analyzed. The performed ANOVA showed statistical differences between the treated cells and the negative control ** *p* < 0.01; *** *p* < 0.001; **** *p* < 0.0001.

All the evaluated compounds caused membrane permeability damage, as shown in Figure 3 and Figure S2 where an intense green fluorescence can be seen in the cytoplasm of the cells. The integrity of the membrane was maintained while the SYTOX green stain was able to enter the cell and link to the DNA.

#### 2.3.3. ROS Production Evaluation

**Figure 4:**
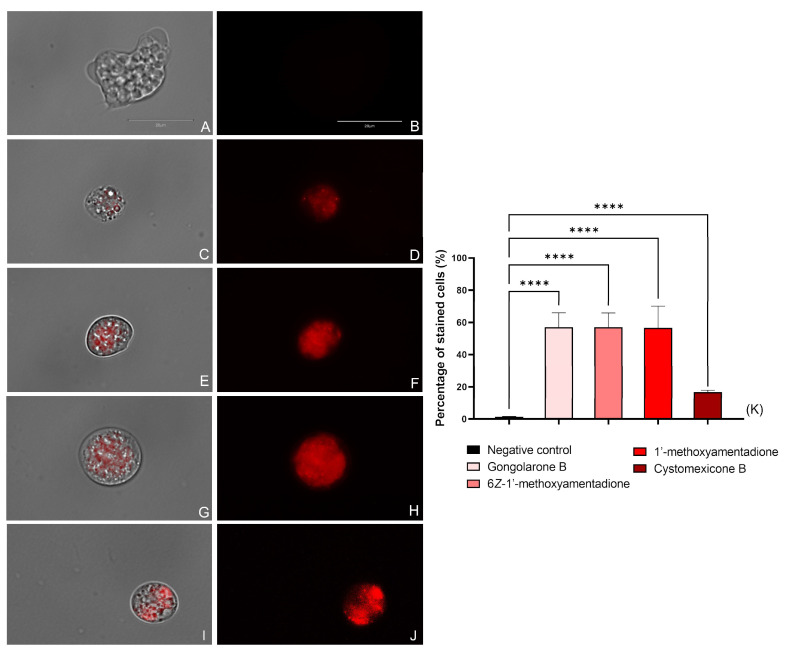
Increased ROS production (red fluorescence) caused by the addition of gongolarone B (**1**) (**C**,**D**), 6*Z-*1′-methoxyamentadione (**2**) (**E**,**F**), 1′-methoxyamentadione (**3**) (**G**,**H**), and cystomexicone B (**6**) (**I**,**J**). Negative control (**A**,**B**) where no fluorescence is observed. Images (×100) are representative of the cell population observed in the performed experiments An EVOS M5000 Cell Imaging System, Life Technologies, Madrid, Spain was used to capture the images. Scale bar: 20 µm. (**K**) The graph includes the percentage of stained cells after the incubation with the CellROX deep red dye. Results are shown as the mean value of three different assays ± SD. An ANOVA was conducted to determine the statistical differences between the negative control and the treated cells **** *p* < 0.0001. Five different images were processed each time.

The *N. fowleri* trophozoites treated with the IC_90_ of the gongolarone B (**1**), 6*Z-*1′-methoxyamentadione (**2**), 1′-methoxyamentadione (**3**), and cystomexicone B (**6**) increased the level of ROS production in comparison with the negative control as can be seen in Figure 4 and Figure S3. Thus, the performed one-way analysis of variance (ANOVA) showed that the differences between the stained amoebae in the non-treated and treated cells were statistically significative, *p*  < 0.0001 (****). Therefore, treatment of the amoebae with the evaluated meroterpenoids induces high levels of oxidative stress.

#### 2.3.4. Mitochondrial Disfunction Evaluation

**Figure 5:**
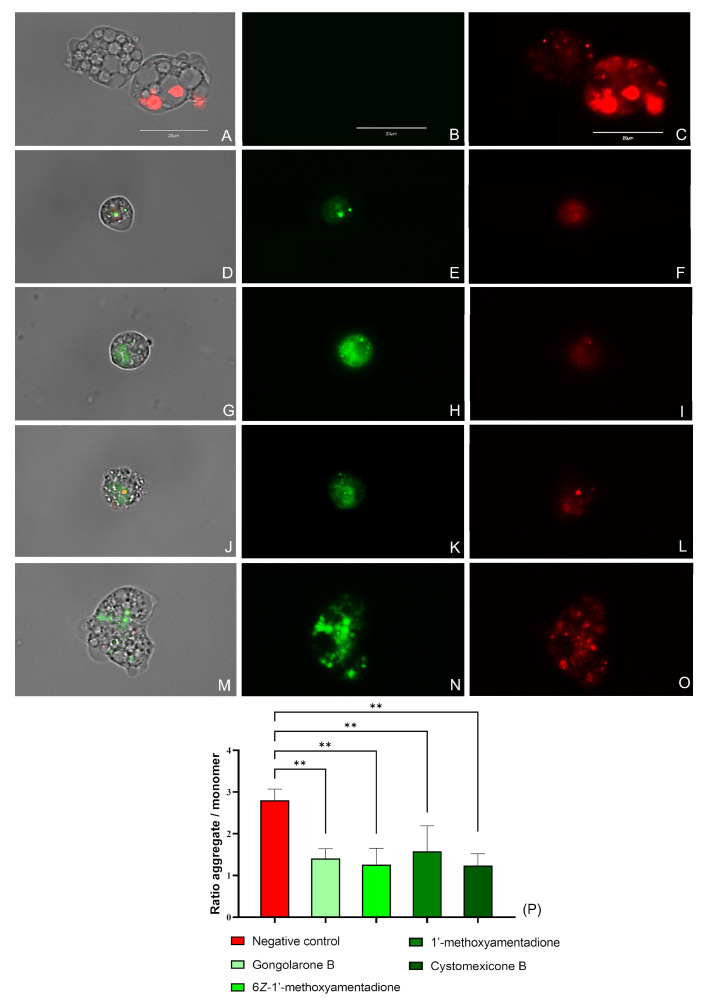
Mitochondrial membrane potential collapse due to the IC_90_ of the gongolarone B (**1**) (**D**–**F**), 6*Z-*1′-methoxyamentadione (**2**) (**G**–**I**), 1′-methoxyamentadione (**3**) (**J**–**L**), and cystomexicone B (**6**) (**M**–**O**). Negative control (**A**–**C**). JC-1 aggregates can be observed in the mitochondria of healthy cells as they emit red fluorescence (red channel: **C**,**F**,**I**,**L**,**O**). Green fluorescence corresponds to treated cells as JC-1 dye is presented in monomers (green channel: **B**,**E**,**H**,**K**,**N**). No green fluorescence is observed in the non-treated amoebae (**C**) Images (×100) are representative of the cell population observed in the performed experiments. An EVOS M5000 Cell Imaging System, Life Technologies, Madrid, Spain was used to capture the images. Scale bar: 20 µm. (**P**) The graph represents the relation between the red and green fluorescences emitted by the JC-1 stain in treated and non-treated trophozoites. Data are depicted as mean value ± SD of three independent experiments. An ANOVA was assessed to determine the statistical differences between the values of the untreated amoebae and the meroterpenoids treated cells; ** *p* < 0.01.

The evaluated compounds cause the mitochondrial membrane potential disruption in *N. fowleri* trophozoites when treated with the IC_90_ of the gongolarone B (**1**), 6*Z-*1′-methoxyamentadione (**2**), 1′-methoxyamentadione (**3**), and cystomexicone B (**6**). As shown in Figure 5 and Figure S4, treated cells emit green fluorescence (Figure 5E,H,K,N) as the JC-1 dye remains in its monomeric form, whereas in the negative control trophozoites (Figure 5B) a bright red fluorescence is visible, corresponding to the “J-aggregates” that forms the stain in healthy mitochondria. No green fluorescence can be observed in the non-treated cells (Figure 5C). In addition, the ratio between the red and the green fluorescence emitted by the JC-1 dye was determined. The performed ANOVA showed that the differences between the calculated ratios of the negative control and the amoebae treated with every meroterpenoid were statistically significant, ** *p* < 0.01.

**Figure 6:**
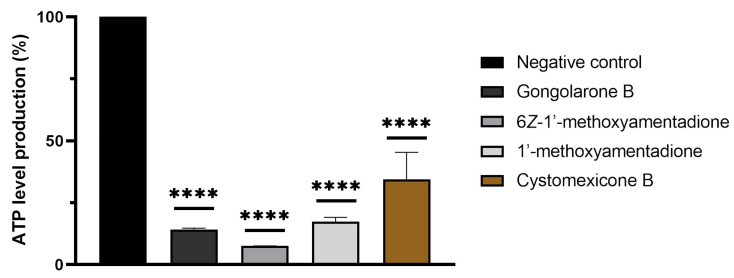
Percentage of relative ATP levels compared to the negative control in *N. fowleri.* The ATP production of a 2 × 10^5^ cells/mL (in treated and non-treated) amoebae concentration was measured. The bars show the average results of three different assays. Error bars represent the standard deviation (SD). The results show a decrease of ATP levels of 85.75 % (gongolarone B (**1**)), 92.49 % (6*Z-*1′-methoxyamentadione (**2**)), 82.62% (1′-methoxyamentadione (**3**)), and 65.59 % (cystomexicone B (**6**)) compared to the negative control (cells without treatment). A Tukey test was carried out to evaluate the statistical differences between the means and the negative control (*p* < 0.0001 [****]).

Moreover, the disfunction of the mitochondria after the treatment of the amoebas with evaluated compounds was confirmed with the measurement of the ATP production. The observed results showed that gongolarone B (**1**), 6*Z-*1′-methoxyamentadione (**2**), 1′-methoxyamentadione (**3**), and cystomexicone B (**6**) decreased the ATP production up to 85.75%, 92.49%, 82.62%, and 65.59%, respectively, when compared to untreated cells (Figure 6).

## 3. Discussion

In the past few years, the study of marine biodiversity has gained enormous attention among the scientific community due to the capacity of these organisms to produce bioactive compounds [17]. In this study, the activity of six meroterpenoids isolated from the brown algae *G. abies-marina* was evaluated against the protozoa *N. fowleri*. Compounds **1**, **2**, **3**, and **6** were active against the two tested strains, showing very similar values in both of them (Table 1). Moreover, the IC_50_ values of the gongolarone B (**1**), 6*Z-*1′-methoxyamentadione (**2**), and 1′-methoxyamentadione (**3**) were lower than the one obtained for the miltefosine (reference drugs for the PAM treatment). However, the CC_50_ of these products was in the same range as the in vitro activity value against the parasite. Nevertheless, in recent years, the chemistry and pharmacological fields have reported several strategies to decrease the toxicity of bioactive compounds. Moreover, the meroterpenoids have a remarkable structural diversity resulting from diverse reactions as condensation, alkylation, oxidation, or reduction [18], so that incorporating different functional groups to this molecular scaffold with the aim of reducing the toxicity could establish an interesting research line in this field. On the other hand, cystomexicone B (**6**) showed less anti-*Naegleria* activity than the other three active meroterpenoids and the two reference drugs. Despite this activity value, the low cytotoxicity shown by cystomexicone B (**6**) makes the selectivity index (SI) of this molecule (SI > 8.5) 2.5-fold higher than the one of the miltefosine (SI = 3.28).

These *G. abies-marina* derivatives where also tested against other protozoa, such as different species of the genus *Acanthamoeba* [16] and *Leishmania*, and against *Trypanosoma cruzi* [19]. Interestingly, despite belonging to the free-living amoebae group, like *N. fowleri*, the most active molecules against all the tested species of *Acanthamoeba* (gongolarones A (**4**) and C (**5**)) showed no anti-*Naegleria* activity. However, the compounds that were active against *N. fowleri* were the same that showed activity against the kinetoplastids *Leishmania* and *T. cruzi*. In fact, *N. fowleri* and the kinetoplastids of these two genera belong to the clade Discicristata, in the Excavata supergroup, a fact that makes them share many traits in the arrangement of the cytoskeleton [20]. On the contrary, *Acanthamoeba* belong to the supergroup of Amoebozoa, hence, despite belonging to the same group (free-living amoebae), the *Naegleria* genus is phylogenetically more separated from *Acanthamoeba* than from the kinetoplastids [21].

Furthermore, the study of the mechanism of cell death determined the presence of different cellular events that are compatible with the apoptosis-like PCD. These events were firstly described in the *Naegleria* genus by Cárdenas-Zúñiga et al. [22]. The incubation of the *N. fowleri* trophozoites with the gongolarone B (**1**), 6*Z-*1′-methoxyamentadione (**2**), 1′-methoxyamentadione (**3**), and cystomexicone B (**6**) induced the appearance of some of these characteristic signs such as the chromatin condensation, plasma membrane permeability damage, mitochondrial damage, and generation and accumulation of ROS. From a clinical point of view, the induction of the apoptosis-like PCD process leads to a cascade of events that ends by triggering an anti-inflammatory response [23]. In contrast, the necrosis process is described as an “accidental” type of cell death characterized by the swelling of organelles, increased cell volume, and the disruption of the plasma membrane, among others. Moreover, necrosis is recognized as a cause of inflammation due to the uncontrolled release of intracellular inflammatory content [24,25]. Hence, the induction of the apoptosis-like PCD by the mentioned meroterpenoids rather than the necrosis could prevent the appearance of an inflammatory response and in consequence the manifestation of undesired side effects.

On the other hand, in previous works of our team, the PCD induction by the amphotericin B has already been reported [26]. Moreover, the in vitro activity and cytotoxicity values of these compound are in a better range than the ones evaluated in this work. Despite this, the low penetration of this drug in the CNS has also been reported [27], in part due to its high molecular weight and its toxicity when administered to humans [28].

**Figure 7:**
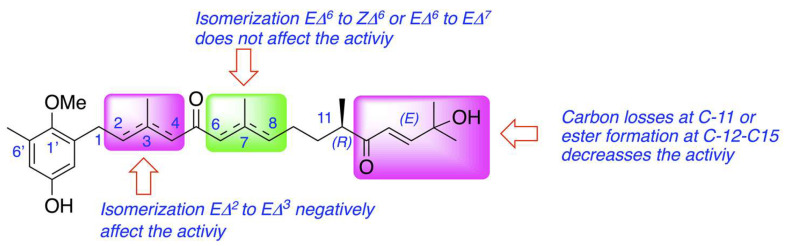
Structure–Activity Relationship analysis of the anti-*Naegleria* activity of compounds **1**–**6**.

From the structural perspective, meroterpenoids **1**–**6** showed double bond isomerization from *E*Δ^2^ to *E*Δ^3^, from *E*Δ^6^ to *Z*Δ^6^, and from *E*Δ^6^ to *E*Δ^7^, as well as modifications at the terminal end of the side chain which involve ester formation and oxidative fragmentation. 6*Z-*1′-methoxyamentadione (**2**) and 1′-methoxyamentadione (**3**) are the most active compounds of this family with IC_50_ values ranging from 13.27 and 20.45 µM. Furthermore, Rodriguez-Expósito et al. (2023) [16] have confirmed using spectroscopic analysis that the evaluated meroterpenoids are lipophilic molecules (with calculated log *p* values between 4.05 and 5.20 (Figure 1)) with a molecular weight of approximately 458 Da which allows them to be good candidates to cross the blood–brain barrier (BBB) [29,30]. Isomerization from *E*Δ^6^ or *Z*Δ^6^ to *E*Δ^7^ do not significatively affect the anti-*Naegleria* activity, showing values of IC_50_ of 18.85 and 21.92 µM. On the other hand, the isomerization of double bond *E*Δ^2^ to *E*Δ^3^ in gongolarone C (**5**) induces a loss of activity. Finally, the ester formation from C-12 to C-15, gongolarone A (**4**), and the oxidative fragmentation at C11-C12, cystomexicone B (**6**), drastically reduces the antiparasitic activity and the toxicity (Figure 7).

## 4. Materials and Methods

### 4.1. Algae Material

*G. abies-marina* was collected off the intertidal zone of the coast of Bajamar, Tenerife, Canary Islands (28°33′15.5″ N 16°20′51.7″ W) [16]. The specimens were cleaned, rinsed, and dried in the dark. The alga was identified by Dr. M. Sansón (Department of Marine Botany of Universidad de La Laguna). A voucher specimen is deposited at the Herbario TFC of SEGAI-ULL under the code 11042019-3.

### 4.2. Extraction, Isolation and Identification of Meroterpenoids **1**–**6**

Dried and ground algal material (233.4 g) was sequentially extracted with dichloromethane (DCM) and ethyl acetate (EtOAc) at room temperature. The filtered organic extracts were combined and evaporated to afford 1.94 g of crude extract. Fractionation by gel filtration chromatography (Sephadex LH-20 column, n-hexane/DCM/methanol (7:2:1)) gave fractions F1–F6. Further chromatographic steps by gel filtration in Sephadex LH-20 (n-hexane/DCM/methanol (3:1:1)) of fraction F6, followed by medium pressure chromatography in a Lobar LiChroprep Si 60 (40–63 μm) column eluted with n-hexane/EtOAc (1:1) and silica gel column (step gradient from CHCl3/EtOAc (4:1) to 100% EtOAc), allowed isolation of cystomexicone B (**6**, 1.39 mg). Pure compounds gongolarone C (**5**, 0.17 mg, rt: 30.2 min), gongolarone B (**1**, 1.75 mg, 34.1 min), *6Z-*1′-methoxyamentadione (**2**, 3.10 mg, 37.1 min), gongolarone A (**4**, 0.48 mg, 39.1 min), and 1′-methoxyamentadione (**3**, 6.22 mg, 42.1 min) were obtained by HPLC of fraction F6.4.3-5 (Phenomenex, Luna 5 μm Silica column, 100 Å, 250 × 10 mm; isocratic n-hexane/EtOAc (3:2), 10 min at 1 mL/min; gradient up to 100% EtOAc, 50 min at 2 mL/min; 100% EtOAc, 5 min, 2 mL/min). Physical properties and NMR data of compounds 1–6 were confirmed with those previously reported [16,31].

### 4.3. Amoebic Strains and Cell Maintenance

The in vitro activity against *N. fowleri* trophozoites was evaluated in two different strains from the American Type Culture Collection (LG Promochem, Barcelona, Spain), ATCC^®^ 30808™ and ATCC^®^ 30215™. Cells were axenically grown in 2% Bactocasitone (*w*/*v*) medium (Thermo Fisher Scientific, Madrid, Spain) at 37 °C. Moreover, 10% (*v*/*v*) of fetal bovine serum (FBS) 0.3 μg/mL of Penicillin G Sodium Salt and 0.5 mg/mL of Streptomycin sulphate (Sigma-Aldrich, Madrid, Spain) were also added to the Bactocasitone medium.

Murine macrophages from the J774A.1 cell line (ATCC^®^ TIB-67) were maintained in in Dulbecco’s Modified Eagle’s medium (DMEM) in order to evaluate the toxicity of the compounds. In addition, 10% (*v*/*v*) FBS and 10 μg/mL gentamicin (Sigma-Aldrich, Madrid, Spain) were added to the growth medium. Cells were grown in a 5% CO_2_ atmosphere at 37 °C.

### 4.4. In Vitro Activity Assays against N. fowleri Trophozoites

For these assays, a colorimetric assay was conducted using the alamarBlue^®^ reagent. Briefly, *N. fowleri* trophozoites were seeded in duplicate in a 96-well microtiter plate. After that, different concentrations of the evaluated meroterpenoids diluted in fresh Bactocasitone were added to the wells. The negative control consisted on the trophozoites incubated with the medium alone. Lastly, the alamarBlue^®^ (Life Technologies, Madrid, Spain) reagent was added. Plates were incubated in slight agitation at 37 °C. After 48 h, the fluorescence of the wells was analyzed in an EnSpire Multimode Plate Reader (Perkin Elmer, Madrid, Spain) using a wavelength of excitation of 570 nm and a wavelength of emission of 585 nm.

In order to calculate the IC_50_ and IC_90_, a nonlinear regression analysis with a 95% confidence limit was performed.

### 4.5. In Vitro Cytotoxicity Assays against Murine Macrophages

For the cytotoxicity assays, the same protocol as detailed in Section 4.4 above was used. Murine macrophages were incubated with serial dilutions of the evaluated compounds at 37 °C in a 5% CO_2_ atmosphere over 24 h. The obtained data were analyzed as described in the previous section in order to calculate the CC_50_.

### 4.6. Mechanism of Cell Death Evaluation

The evaluation of the programmed cell death induction by the meroterpenoids was performed in the ATCC^®^ 30808™ *N. fowleri* trophozoites. Cells were incubated over 24 h with the IC_90_ (Table 1) of the compounds at 37 °C. In these experiments, the presence of different metabolic events which are characteristics of the programmed cell death process were evaluated. For this, five different apoptosis events marker kits (the Hoechst 33342/Propidium Iodide, SYTOX™ Green kit, CellROX^®^ Deep Reagent, JC-1 Mitochondrial Membrane Potential Detection Kit, and the Celltiter-Glo^®^ Luminescent Cell Viability Assay) were used according to manufacturer’s instructions. Furthermore, the percentage of stained cells was determined after incubating the treated and non-treated cells with the stains.

#### 4.6.1. Chromatin Condensation Detection

For the chromatin condensation assay, the Hoechst 33342 and the PI (Life Technologies, Madrid, Spain) were used. The images were captured in the EVOS™ M5000 Imaging System (Invitrogen by Thermo Fisher Scientific). A concentration of 5 × 10^5^ cell/mL was seeded and incubated with compounds over 24 h.

Three different cell population can be distinguished with this kit. Firstly, healthy cells will barely show blue fluorescence whereas in the presence of condensed chromatin an intense blue fluorescence corresponding to the Hoechst 33342 stain will be observable. Finally, dead cells will show red fluorescence due to the linkage between the nucleus and the PI.

#### 4.6.2. Plasma Membrane Permeability

The evaluation of the damage in the cell membrane permeability was conducted with the SYTOX Green assay (Life Technologies, Madrid, Spain). Amoebae were incubated with the molecules over 24 h at 37 °C. An EVOS™ M5000 Imaging System (Invitrogen by Thermo Fisher Scientific) was used to observe the trophozoites. Cells with compromised plasma membrane permeability show bright green fluorescence as the stain reaches the nucleus. However, no fluorescence is observed in negative control cells.

#### 4.6.3. Reactive Oxygen Species (ROS) Production

The determination of ROS generation was carried out using the CellROX Deep Red fluorescent assay (Thermo Fisher Scientific) following manufacturer’s instructions. Briefly, *N. fowleri*’s trophozoites were incubated with compounds over 24 h at 37 °C. After that, the CellROX stain was added and incubated with the cells for 30 min in the dark. Finally, the amoebas were observed in an EVOS™ M5000 Imaging System (Invitrogen by Thermo Fisher Scientific). The CellROX Deep Red dye penetrates the cells and emits no fluorescence in its reduced state; however, it exhibits a bright fluorescence upon oxidation by ROS.

#### 4.6.4. Mitochondrial Function Analysis

The mitochondrial damage was evaluated using two different reagents. Firstly, the JC-1 Mitochondrial Membrane Potential Detection Kit (Cayman Chemicals Vitro SA, Madrid, Spain) was used to assess the failure of the mitochondrial membrane potential. Cells were incubated with the compounds at 37 °C to finally add the JC-1 reagent after 24 h. The JC-1 dye is presented in the mitochondria of untreated cells in aggregate form and emits red fluorescence. However, when the membrane potential decreases (unhealthy cells) the dye is presented in monomers, emitting green fluorescence. Images were obtained with an EVOS™ M5000 Imaging System (Invitrogen by Thermo Fisher Scientific).

On the other hand, the measurement of the ATP production was also assessed in order to confirm the mitochondrial damage in the amoebae. For this, the Celltiter-Glo^®^ Luminescent Cell Viability Assay (Promega Biotech Ibérica, Madrid, Spain) was used. The emitted luminescence, which is proportional to the ATP levels, was measured in an EnSpire^®^ Multimode Plate Reader (Perkin Elmer, Madrid, Spain). Experiments were performed in triplicate. In both treated and untreated cells, the ATP production of a concentration of 2 × 10^5^ cells/mL was measured.

### 4.7. Statistical Analysis

The in vitro IC_50_, IC_90_, and CC_50_ of the compounds were determined by a non-linear regression with a 95% confidence limit. The SigmaPlot 12.0 software (Systat Sofware Inc., London, UK) was used to perform the data analysis using a paired two-tailed *t*-test. Values of *p* < 0.05 were considered significant. The data represent the mean value and the SD of the conducted three different experiments.

In the PCD assays, the number of stained and unstained cells was measured after the performance of the experiments. Regarding the JC-1 Mitochondrial Membrane Potential assays, the ratio between the emitted red fluorescence (at 595 nm) and green fluorescence (at 535 nm) was determined. For each kit, images (×40) with at least 80 cells were selected and analyzed. These data were obtained in the EVOS™ M5000 Software (Invitrogen by Thermo Fisher Scientific). The graphs illustrate the mean value ± SD of three different tests. Finally, a one-way analysis of variance (ANOVA) was assessed to determine the statistical differences between the treated cells and the negative control amoebae, ** *p* < 0.01; *** *p* < 0.001; **** *p* < 0.0001 significant differences.

## 5. Conclusions

In this study, the activity of six meroterpenoids obtained from the brown algae *Gongolaria abies-marina* was studied against two different strains of *N. fowleri*. Results showed that compounds **1**, **2**, and **3** were the most active molecules, with lower IC_50_ values than the reference drug, miltefosine. However, the cystomexicone B (**6**) showed the highest selectivity index (>8.5) due to its low toxicity. Moreover, the determination of cell death type showed that these compounds induce the appearance of cellular events compatible with the programmed cell death process induced by amphotericin B. In conclusion, the algae *G. abies-marina* can be considered as a source of compounds to treat the PAM.

## Figures and Tables

**Table 1:** Inhibitory concentration 50 (IC_50_, µM) and 90 (IC_90_, µM) against two different strains of *N. fowleri* and cytotoxic concentration 50 (CC_50_, µM) and 90 (CC_90_, µM) of the evaluated compounds. The values of the amphotericin B and miltefosine are also represented as reference drugs to treat the PAM.

Compounds	IC_50_ ATCC^®^ 30808	IC_50_ ATCC^®^ 30215	CC_50_ ATCC^®^ TIB-67	IC_90_ ATCC^®^ 30808	IC_90_ ATCC^®^ 30215	CC_90_ ATCC^®^ TIB-67
Gongolarone B (**1**)	21.92 ± 1.60	18.85 ± 0.94	25.62 ± 4.16	58.01 ± 1.73	68.26 ± 1.73	72.86 ± 5.94
6*Z*-1′Methoxyamentadione (**2**)	20.45 ± 4.07	13.27 ± 0.96	26.56 ± 4.03	55.30 ± 8.28	44.17 ± 14.89	67.15 ± 6.32
1′Methoxyamentadione (**3**)	19.86 ± 5.27	16.51 ± 0.96	26.19 ± 2.25	64.86 ± 6.94	59.74 ± 20.19	68.67 ± 7.46
Gongolarone A (**4**)	>100	-	>400	-	-	-
Gongolarone C (**5**)	>100	-	>400	-	-	-
Cystomexicone B (**6**)	58.91 ± 10.07	58.07 ± 5.85	>500	206.37 ± 16.62	259.73 ± 41.09	>500
Amphotericin B	0.12 ± 0.03	0.16 ± 0.03	>200	-	-	-
Miltefosine	38.74 ± 4.23	81.57 ± 7.23	127.89 ± 8.85	-	-	-

## Data Availability

Data is contained within the article and Supplementary Material.

## Supplementary Materials

The following supporting information can be downloaded at: https://www.mdpi.com/article/10.3390/ph16071010/s1, Figure S1: *Naegleria fowleri* trophozoites incubated with IC_90_ of the evaluated compounds for 24 h (**D**–**O**). Negative control (**A**–**C**), gongolarone B (**1**) (**D**–**F**), 6*Z*–1′–methoxyamentadione (**2**) (**G**–**I**), 1′–methoxyamentadione (**3**) (**J**–**L**), and cystomexicone B (**6**) (**M**–**O**). Hoechst channel (**B**, **E**, **H**, **K**, **N**), and propidium iodide channel (**C**, **F**, **I**, **L**, **O**). Figure S2: Plasma membrane permeability assay in *Naegleria fowleri* trophozoites. Negative control (**A** and **B**), gongolarone B (**1**) (**C** and **D**), 6*Z*–1′–methoxyamentadione (**2**) (**E** and **F**), 1′–methoxyamentadione (**3**) (**G** and **H**), and cystomexicone B (**6**) (**I** and **J**). Figure S3: Incubation of the CellROX Deep Red stain with cells after treating them with the IC_90_ of gongolarone B (**1**) (**C** and **D**), 6*Z*–1′–methoxyamentadione (**2**) (**E** and **F**), 1′–methoxyamentadione (**3**) (**G** and **H**), and cystomexicone B (**6**) (**I** and **J**). Negative control (**A** and **B**). Figure S4: Alterations in the mitochondrial membrane potential after incubating the *Naegleria fowleri* cells with the IC_90_ of the gongolarone B (**1**) (**D**–**F**), 6*Z*–1′–methoxyamentadione (**2**) (**G**–**I**), 1′–methoxyamentadione (**3**) (**J**–**L**), and cystomexicone B (**6**) (**M**–**O**). Negative control (**A**–**C**). Red channel (**C**, **F**, **I**, **L**, **O**). Green channel (**B**, **E**, **H**, **K**, **N**).
