# Supplementary material for: Marine Meroterpenoids Isolated from Gongolaria abies-marina Induce Programmed Cell Death in Naegleria fowleri
Source: Pharmaceuticals (Basel). 2023 Jul 17;16(7):1010. doi: 10.3390/ph16071010 (PMC10384572; doi:10.3390/ph16071010)
Supplement: Supplementary file 1 [file pharmaceuticals-16-01010-s001.zip › pharmaceuticals-2478891-supplementary.pdf]

# Supporting Information

# Marine meroterpenoids isolated from *Gongolaria abies-marina* induce programmed cell death in *Naegleria fowleri*

Iñigo Arberas-Jiménez[1,2], Rubén L. Rodríguez-Expósito[1,2], Desirée San Nicolás-Hernández[1,2], Javier Chao-Pellicer[1,2,3], Ines Sifaoui[1,2], Ana R. Díaz-Marrero[4,5]*, José J. Fernández[4,6]*, José E. Piñero[1,2,3]*, Jacob Lorenzo-Morales[1,2,3]*

[1] Instituto Universitario de Enfermedades Tropicales y Salud Pública de Canarias (IUETSPC), Universidad de La Laguna (ULL), Avda. Astrofísico Fco. Sánchez S/N, 38206 La Laguna, Tenerife, Spain

[2] Departamento de Obstetricia y Ginecología, Pediatría, Medicina Preventiva y Salud Pública, Toxicología, Medicina Legal y Forense y Parasitología, Universidad de La Laguna (ULL), 38206 La Laguna, Tenerife, Spain

[3] Consorcio Centro de Investigación Biomédica En Red (CIBER) de Enfermedades Infecciosas (CIBERINFEC), Instituto de Salud Carlos III, 28006 Madrid, Spain

[4] Instituto Universitario de Bio-Orgánica Antonio González, Universidad de La Laguna (ULL), Avda. Astrofísico Fco. Sánchez 2, 38206 La Laguna, Spain

[5] Instituto de Productos Naturales y Agrobiología (IPNA), Consejo Superior de Investigaciones Científicas (CSIC), Avda. Astrofísico Fco. Sánchez 3, 38206 La Laguna, Tenerife, Spain

[6] Departamento de Química Orgánica, Universidad de La Laguna (ULL), Avda. Astrofísico Fco. Sánchez 3, 38206 La Laguna, Tenerife, Spain

* Correspondence: adiazmar@ipna.csic.es (A.R.D.-M.); jpinero@ull.edu.es (J.E.P); jjfercas@ull.edu.es (J.J.F.); jmlorenz@ull.edu.es (J.L.-M.)

# Table of contents


| Index | |
|---|---|
| **Figure S1.** Chromatin condensation and PI assays in treated *Naegleria fowleri* trophozoites (x40). | **S3** |
| **Figure S2.** Plasma membrane permeability evaluation with the SYTOX green dye (x40). | **S4** |
| **Figure S3.** Evaluation of the ROS production after the treatment of *Naegleria fowleri* with the evaluated meroterpenoids (x40). | **S5** |
| **Figure S4.** Mitochondrial membrane potential decrease induction by meroterpenoids in *Naegleria fowleri* trophozoites (x100). | **S6** |

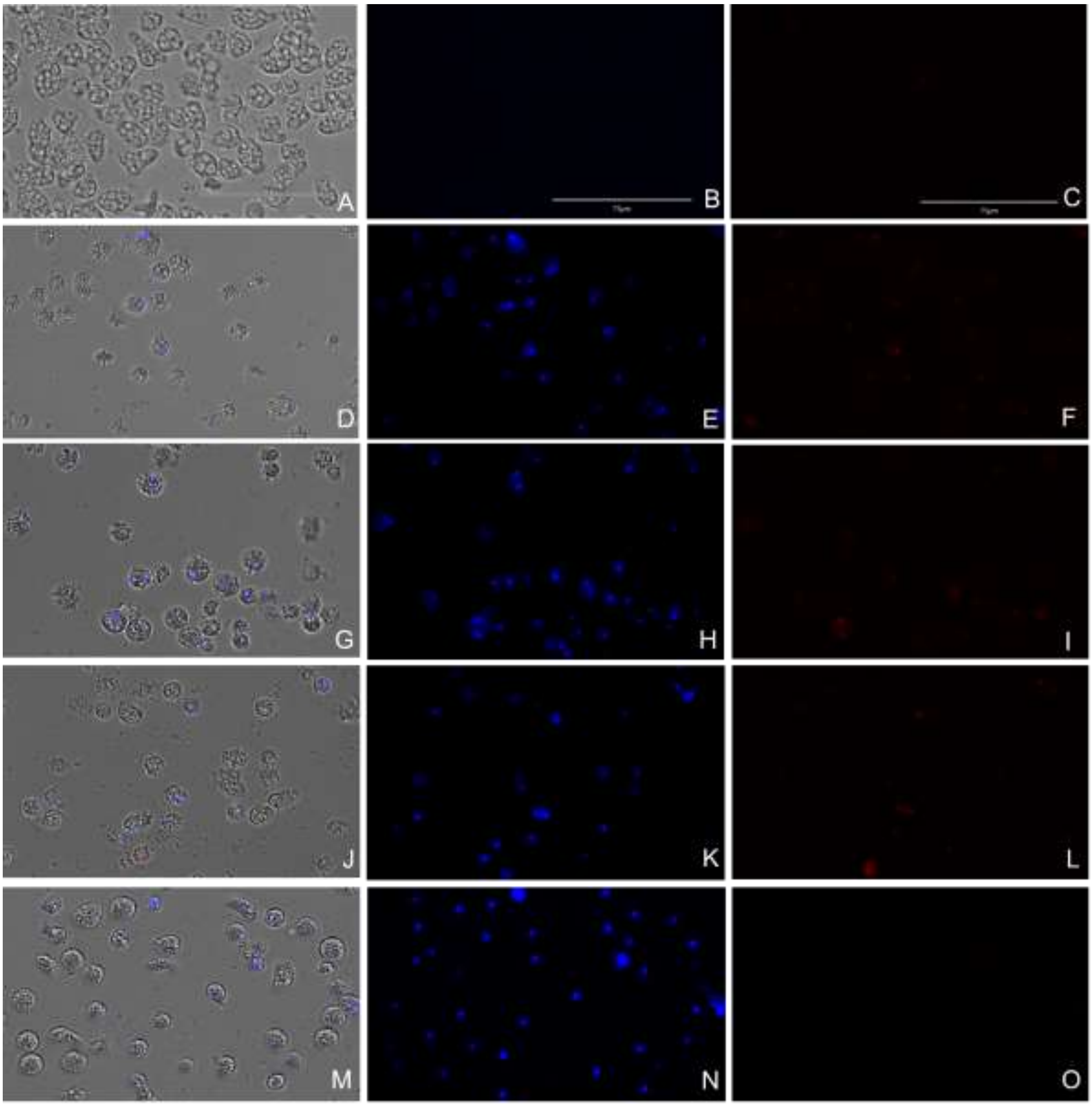


**Figure S1**. *Naegleria fowleri* trophozoites incubated with $IC_{90}$ of the evaluated compounds for 24 h (**D**-**O**). Negative control (**A**-**C**), gongolarone B (**1**) (**D**-**F**), 6*Z*-1′-methoxyamentadione (**2**) (**G**-**I**), 1′-methoxyamentadione (**3**) (**J**-**L**) and cystomexicone B (**6**) (**M**-**O**). Hoechst channel (**B,E,H,K,N**), and propidium iodide channel (**C,F,I,L,O**). Images (40×) are representative of the cell population observed in the performed experiments. Images were obtained using an EVOS M5000 Cell Imaging System (Life Technologies, Madrid, Spain). Scale bar: 75 μm.

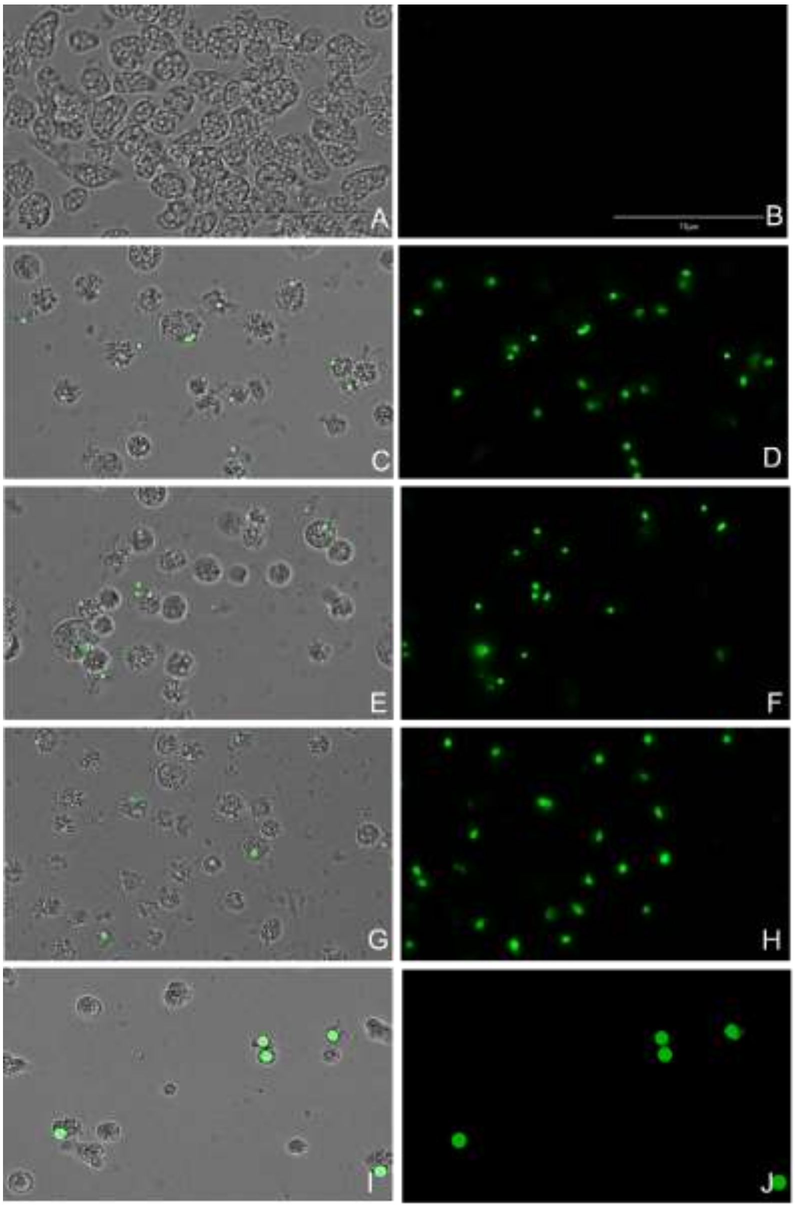


**Figure S2**. Plasma membrane permeabilty assay in *Naegleria fowleri* trophozoites. Negative control (**A,B**), gongolarone B (**1**) (**C**-**D**), 6*Z*-1'-methoxyamentadione (**2**) (**E**-**F**), 1'-methoxyamentadione (**3**) (**G**-**H**) and cystomexicone B (**6**) (**I**-**J**). Images (x40) are representative of the cell population observed in the performed experiments. Images were obtained using an EVOS M5000 Cell Imaging System (Life Technologies, Madrid, Spain). Scale bar: 75 μm.

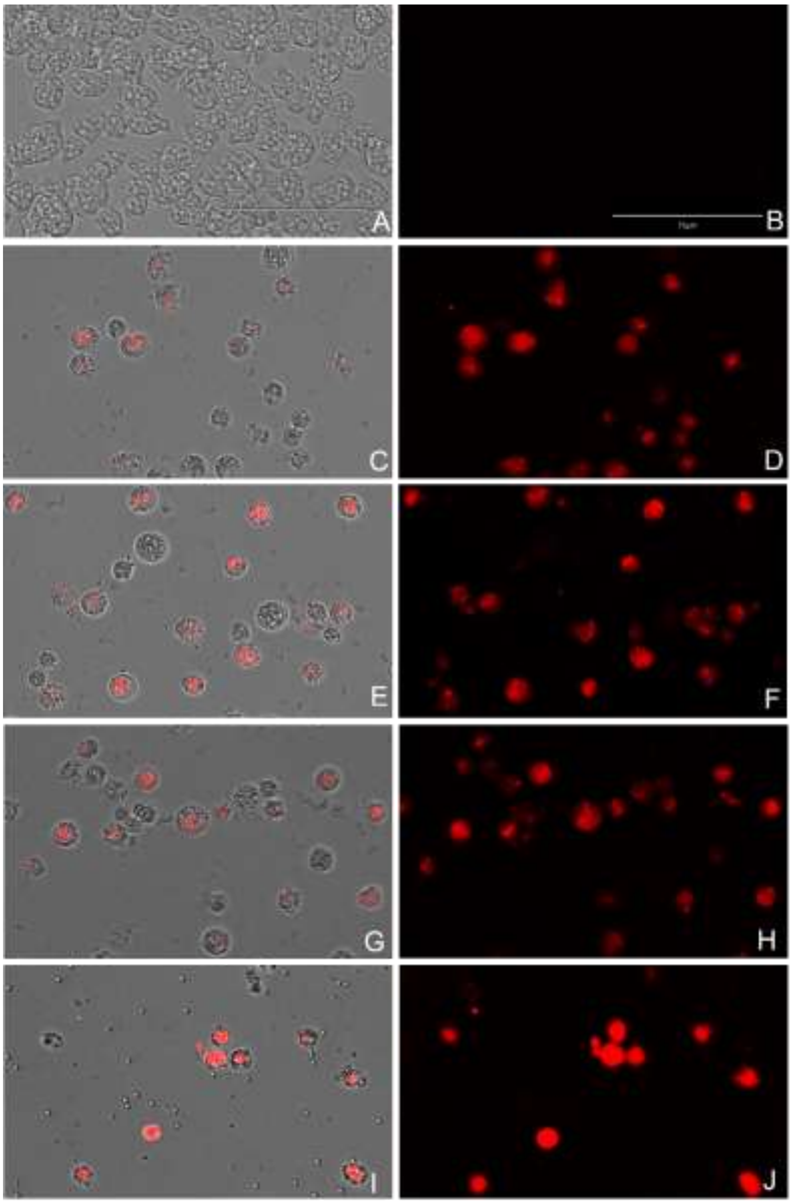


**Figure S3** Incubation of the CellROX Deep Red stain with cells after treating them with the $IC_{90}$ of gongolarone B (**1**) (**C**-**D**), 6*Z*-1′-methoxyamentadione (**2**) (**E**-**F**), 1′-methoxyamentadione (**3**) (**G**-**H**) and cystomexicone B (**6**) (**I**-**J**). Negative control (**A,B**). Images (x40) are representative of the cell population observed in the performed experiments. Images were obtained using an EVOS M5000 Cell Imaging System (Life Technologies, Madrid, Spain). Scale bar: 75 μm.

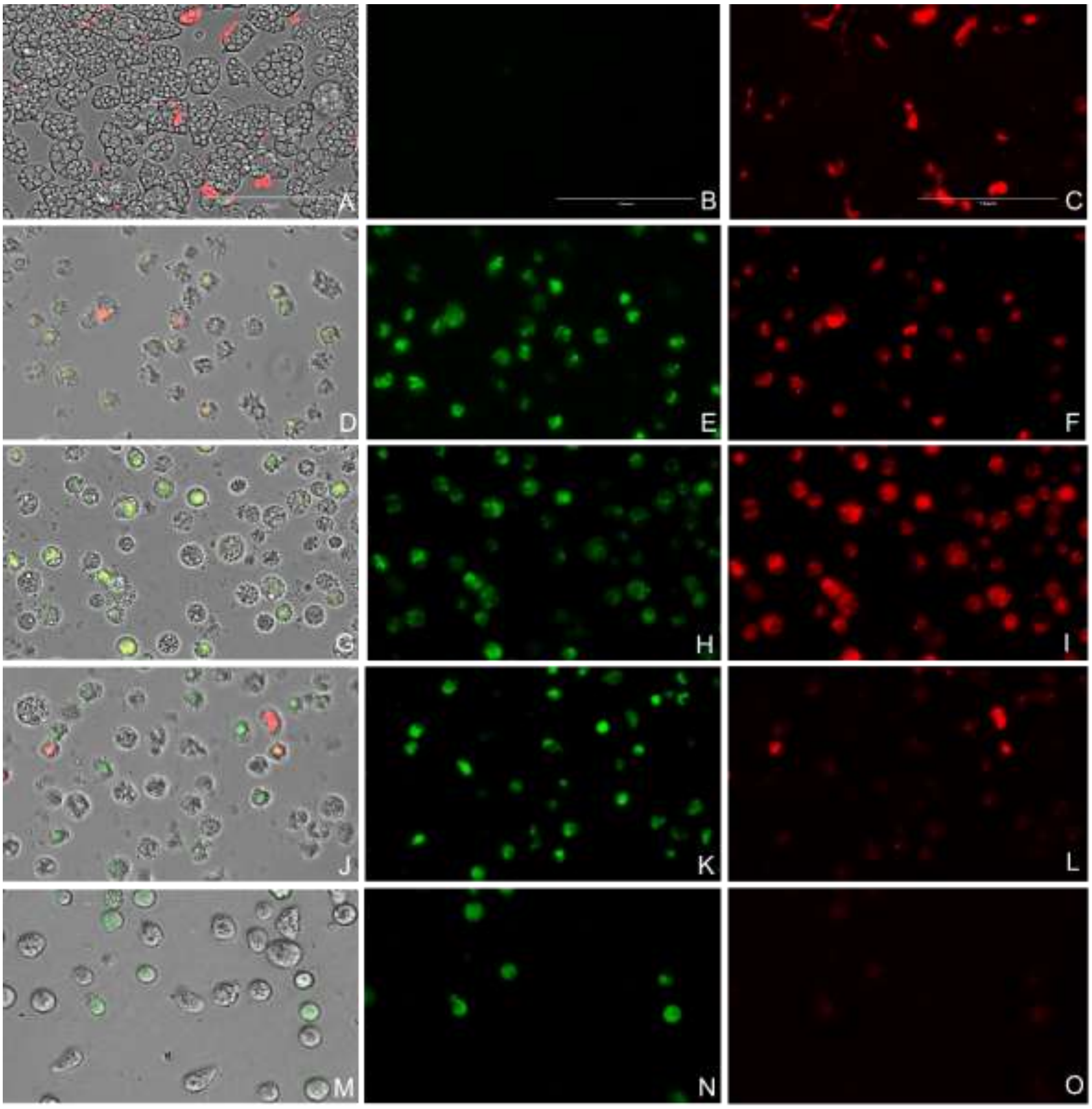


**Figure S4.** Alterations in the mithonodrial membrane potential after incubating the *Naegleria fowleri* cells with the $IC_{90}$ of the gongolarone B (**1**) (**D**-**F**), 6*Z*-1'-methoxyamentadione (**2**) (**G**-**I**), 1'-methoxyamentadione (**3**) (**J**-**L**) and cystomexicone B (**6**) (**M**-**O**). Negative control (**A**-**C**). Red channel (**C,F,I,L,O)**. Green channel (**B,E,H,K,N**). Images (x40) are representative of the cell population observed in the performed experiments. Images were obtained using an EVOS M5000 Cell Imaging System (Life Technologies, Madrid, Spain).
